# The hidden threat: understanding anti-PF4 disorders without proximate heparin exposure

**DOI:** 10.3389/fimmu.2026.1828051

**Published:** 2026-09-07

**Authors:** Zhiyan Liu, Yan Li, Liang Ma, Xin Wang, Yimin Cui

**Affiliations:** 1 Institute of Clinical Pharmacology, Peking University First Hospital, Beijing, China; 2 Department of Cardiology, The First Affiliated Hospital of Xinjiang Medical University, Urumqi, Xinjiang, China; 3 Clinical Trial Research Center, Beijing Hospital, Beijing, China

**Keywords:** anti-PF4 disorders, heparin, HIT, platelet factor 4, thrombosis

## Abstract

Heparin-induced thrombocytopenia (HIT) is now recognized as one subset of a broader group of disorders: anti-platelet factor 4 (anti-PF4) disorders. This review provides an updated framework for anti-PF4 immunothrombosis without proximate heparin exposure—a clinical syndrome with phenotypic and laboratory features resembling classic HIT but with no recent heparin use (no heparin exposure within 14 days before onset). The core characteristics are a ≥50% platelet count decrease from baseline or an absolute platelet count <100×10^9^/L, often associated with elevated arterial or venous thrombosis risk. Historically, these heparin-independent syndromes encompass spontaneous HIT-like syndrome, vaccine-induced immune thrombotic thrombocytopenia (VITT/TTS), and autoimmune HIT (aHIT)—notably, nearly all aHIT cases are initiated by prior heparin exposure before progressing to sustained heparin-independent platelet-activating antibodies. This review exclusively focuses on patients with no heparin exposure within 14 days of symptom onset, including: (1) patients with prior heparin exposure >14 days ago whose antibodies have evolved heparin-independent pathogenicity (aHIT), (2) patients with truly spontaneous HIT-like syndrome without any prior heparin, and (3) VITT. For aHIT specifically, prior heparin exposure is the initiating event in >95% of cases, but the antibodies become heparin-independent over time; thus these patients fall within our 14-day window of interest when presenting with delayed-onset thrombocytopenia beyond the acute heparin exposure phase. Pathogenesis centers on PF4-driven abnormal immune responses: PF4 forms complexes with polyanions (von Willebrand factor [VWF], pathogen surface molecules, etc.), inducing pathogenic antibodies that activate platelets via FcγRIIA, creating a self-amplifying “immune activation–thrombosis” cycle. Diagnosis requires combined clinical, laboratory, and imaging evaluation to distinguish from other thrombocytopenia-with-thrombosis disorders. Current treatment includes non-heparin anticoagulation plus high-dose IVIG for heparin-independent forms. Future research should validate diagnostic criteria, biomarkers, and targeted therapies.

## 1 Introduction

Heparin-induced thrombocytopenia (HIT) is an immune-mediated disease triggered by heparin exposure, characterized by decreased platelet count and increased risk of thrombosis (1). The pathogenesis of classic HIT involves the production of anti-platelet factor 4/heparin (PF4/H) antibodies, which bind to PF4/H complexes and activate platelets via FcγRIIA receptors, ultimately leading to platelet consumption and thrombosis (2). However, clinical observations have identified a group of syndromes with clinical manifestations highly similar to HIT but without a history of heparin exposure, referred to as spontaneous HIT-like syndrome, atypical HIT, or autoimmune HIT (aHIT) (3, 4). A critical distinction must be made: spontaneous HIT-like syndrome and VITT arise without any prior heparin exposure, whereas aHIT is typically (>95%) a sequela of antecedent heparin administration, in which high-titer anti-PF4/heparin antibodies transform into persistent heparin-independent pathogenic clones over time. All three entities share the phenotype of thrombocytopenia and thrombosis driven by heparin-independent anti-PF4 antibodies, unifying them as anti-PF4 disorders presenting without proximate heparin exposure (defined as no heparin within 14 days of symptom onset). True aHIT *de novo* without any prior heparin exposure is exceptionally rare (<5%) and, when encountered, is managed identically to aHIT with antecedent heparin sensitization. With in-depth research, heparin-free HIT-like syndromes have also been found in neonates, patients with specific underlying diseases (such as infections and autoimmune diseases), and those who have undergone specific surgical procedures (5). Especially during the coronavirus disease 2019 (COVID-19) pandemic, the discovery of vaccine-induced immune thrombotic thrombocytopenia (VITT) has significantly expanded the understanding of such syndromes (6). This article aims to provide a review of anti-PF4 disorders, systematically elaborate the PF4-VWF pathogenic complex model proposed by our research team, stratified management for neonatal, post-surgical, infection-associated and autoimmune disease complicated subgroups, and provides minimal discussion of monoclonal anti-PF4 antibody pathogenesis in refractory aHIT.

Unlike the recent authoritative overview by Warkentin and Greinacher (7), which comprehensively addresses all anti-PF4 disorders including classic heparin-dependent HIT, this review deliberately restricts its scope to patients without proximate heparin exposure (defined as no heparin within 14 days prior to symptom onset). This focused population poses unique diagnostic challenges, as the absence of a clear heparin trigger often leads to missed or delayed diagnosis. Moreover, we provide a conceptual framework that explicitly distinguishes three pathophysiologically distinct pathways to heparin-independent anti-PF4 immunothrombosis: (1) heparin-primed affinity maturation (aHIT), (2) non-pharmacologic polyanion-triggered *de novo* immune response (spontaneous HIT-like syndrome), and (3) adenoviral antigen-driven molecular mimicry (VITT). This tripartite framework—not articulated in prior reviews—offers a practical clinical heuristic for differentiating these entities despite their overlapping laboratory phenotypes.

## 2 Classification and general features of anti-PF4 disorders

### 2.1 Terminology and classification framework

According to current literature (1, 2, 8–20), anti-PF4 disorders are classified into several distinct entities based on trigger, heparin dependence, and laboratory profile (**Table 1**).

**Table 1:** Representative terms and background of anti-PF4 disorders.

Term	Year	Key background	Priming trigger	Monoclonal antibody involvement
Spontaneous HIT-like syndrome	2008	Divided into surgical subtype (post-orthopedic/cardiac surgery) and medical subtype (post-infection); no heparin exposure at any time point.	Surgery, trauma, infection (no heparin)	Rare
Autoimmune HIT (aHIT)	2011	Distinguishes heparin-dependent vs. heparin-independent anti-PF4 diseases; >95% of cases are primed by prior heparin exposure, with high-titer antibodies evolving to persistent heparin-independent platelet-activating capacity; addresses overdiagnosis of classic HIT.	Prior heparin exposure (mandatory for typical aHIT)	Frequent in refractory cases — monoclonal anti-PF4 clones drive sustained platelet activation
Vaccine-induced immune thrombotic thrombocytopenia (VITT)	2021	Associated with adenovirus-vectored COVID-19 vaccines (ChAdOx1, Ad26.COV2.S); ISTH issued interim guidelines.	Adenoviral vector vaccine	Rare
Thrombotic thrombocytopenia syndrome (TTS)	2021	Descriptive term for thrombosis + thrombocytopenia; used alongside VITT.	Adenoviral vector vaccine	Rare

#### 2.1.1 Classic HIT

Heparin-dependent, anti-PF4/heparin antibodies, platelet activation enhanced by low heparin and inhibited by high heparin, and onset 5–14 days after heparin exposure (1, 2).

#### 2.1.2 Autoimmune HIT

Over 95% of aHIT cases are primed by prior heparin exposure, which induces robust production of high-titer anti-PF4/heparin IgG antibodies. A subset of these antibodies evolves persistent heparin-independent platelet-activating capacity even after complete heparin discontinuation, driving sustained thrombocytopenia and recurrent thrombosis without ongoing heparin stimulation. True *de novo* aHIT with zero prior heparin contact accounts for less than 5% of all aHIT cases and represents an exceptional clinical phenotype. Circulating antibody mixtures display heterogeneous responsiveness to exogenous heparin; emerging evidence confirms refractory, long-lasting aHIT is predominantly mediated by monoclonal anti-PF4 antibody clones, which explains its poor response to anticoagulation monotherapy alone. Secondary inflammatory triggers (major surgery, severe infection) further amplify the persistence of these pathogenic antibodies after initial heparin sensitization (8, 9, 18).

#### 2.1.3 Spontaneous HIT-like syndrome

Occurs without heparin or vaccine triggers; associated with infection, surgery, trauma, or inflammation; serology and functional tests confirm heparin-independent anti-PF4 immunothrombosis (10–13).

#### 2.1.4 VITT and VITT-like disorders

Occurs 5–30 days (up to 42 days for isolated DVT/PE) after adenoviral-vector COVID-19 vaccination; PF4-dependent but heparin-inhibited platelet activation; thrombocytopenia; major thrombosis (especially cerebral venous sinus or splanchnic veins); markedly elevated D-dimer, and positive anti-PF4 ELISA (14–18).

#### 2.1.5 Other anti-PF4 antibody positivity without platelet activation (non-pathogenic)

Commonly detected in infections, autoimmune diseases, and post-surgery, these antibodies do not induce platelet activation in functional assays and are not associated with thrombotic risk (2, 19, 20).

This review focuses on entities (2)-(4), collectively referred to as anti-PF4 disorders without proximate heparin exposure. The distinction between these subtypes is not merely semantic; it carries critical diagnostic and therapeutic implications, as summarized in **Table 2**.

**Table 2:** Laboratory and functional assay differences among anti-PF4 disorders.

Feature	Classic HIT	Autoimmune HIT (aHIT)	Spontaneous HIT-like syndrome	VITT / VITT-like
Heparin exposure	Yes (recent, proximate)	Prior heparin exposure (heparin-primed; may be remote at symptom onset)	No (never exposed)	No (vaccine/infection trigger)
PF4-dependent ELISA	Positive (heparin-dependent)	Positive (heparin-independent; high titer)	Positive (heparin-independent)	Positive (heparin-independent; high titer)
Rapid immunoassay (e.g., HIT IgG)	Usually positive	May be false negative (heparin-independent antibodies not detected by standard rapid assays)	May be false negative	Often false negative
Functional assay – low heparin	Enhanced activation	Variable / sustained activation — monoclonal antibody clones show persistent activation independent of heparin concentration	Variable	Usually no enhancement
Functional assay – high heparin	Inhibition	Variable / partial inhibition — monoclonal clones may remain activatory	Variable	Inhibition
PF4-enhanced assay (PF4-SRA/PIPA)	Positive	Positive	Positive	Required for diagnosis
Monoclonal antibody signature	Absent	Frequently present in refractory cases — persistent strong platelet activation across all heparin concentrations	Absent	Rare
D-dimer	Elevated	Elevated	Elevated	Markedly elevated (>10× ULN)
Fibrinogen	Normal or elevated	Normal or elevated	Normal or elevated	Low or normal
Thrombosis sites	DVT, PE, arterial	DVT, PE, arterial (recurrent/persistent)	DVT, PE, arterial	Cerebral venous sinus, splanchnic
Treatment	Non-heparin anticoagulation	Non-heparin anticoagulation + IVIG (mandatory for moderate-severe)	Non-heparin anticoagulation + IVIG	Non-heparin anticoagulation + IVIG

ULN, upper limit of normal; SRA, serotonin release assay; PIPA, PF4-induced platelet activation assay.

### 2.2 Pathogenesis

#### 2.2.1 Established core mechanisms

1) PF4, a cationic chemokine, forms complexes with polyanions (heparin, bacterial polysaccharides, nucleic acids, or glycosaminoglycans). 2) These complexes expose neoepitopes that trigger pathogenic anti-PF4 IgG antibodies. 3) Antibodies activate platelets via the FcγRIIA receptor, leading to platelet aggregation, release of procoagulant microparticles, and endothelial injury. 4) Downstream thromboinflammation drives overt thrombosis. This is well established for classic HIT and increasingly recognized in aHIT and VITT (1, 2)s.

#### 2.2.2 Hypothetical/investigational mechanisms

Based on previous work from our group (21), we propose a PF4-VWF complex hypothesis for non-heparin anti-PF4 immunothrombosis (e.g., VITT, infection-related cases): PF4 binds VWF to form immunogenic complexes in the absence of heparin. These complexes expose neoepitopes that trigger pathogenic anti-PF4 IgG antibodies. This model remains hypothetical and requires validation in formal experimental studies. It is not yet regarded as an established mechanism of anti-PF4 disorders, and several knowledge gaps persist, including the precise stoichiometry of PF4-VWF complex formation, the identification of the specific VWF domains involved, and the relative contribution of this pathway versus other endogenous polyanions (e.g., polyphosphates, nucleic acids) in different clinical contexts.

Proposed translational implications of the PF4-VWF hypothesis for clinical practice: If validated, the PF4-VWF complex model would provide a unified mechanistic explanation for why certain infections (e.g., Gram-negative sepsis with high LPS burden), major surgeries (with extensive endothelial injury releasing VWF), and inflammatory states (with upregulated VWF secretion) predispose to anti-PF4 immunothrombosis even without heparin. This framework generates three testable clinical predictions: (1) plasma VWF levels or VWF-PF4 complex levels may serve as biomarker candidates for risk stratification in surgery/infection patients with thrombocytopenia; (2) conditions characterized by marked VWF elevation (e.g., critical illness, sepsis, ECMO) may warrant heightened vigilance for heparin-independent anti-PF4 disorders; (3) therapeutic strategies targeting the PF4-VWF interaction interface could offer a novel approach distinct from FcγRIIA blockade. Although these implications remain speculative, they define a concrete research agenda that distinguishes our review from prior comprehensive overviews by providing hypothesis-driven clinical directions rather than purely descriptive classification.

#### 2.2.3 Key mechanistic differences between classic HIT and heparin-independent anti-PF4 disorders

While classic HIT and the heparin-independent anti-PF4 disorders (aHIT, spontaneous HIT-like syndrome, VITT) share the core triad of anti-PF4 antibodies, FcγRIIA-mediated platelet activation, and thromboinflammatory sequelae, fundamental mechanistic distinctions exist. In classic HIT, PF4 undergoes a conformational change upon binding to heparin, exposing neoepitopes that trigger pathogenic antibody production. Critically, the resulting anti-PF4/heparin antibodies are heparin-dependent for platelet activation: low concentrations of heparin (0.1-0.3 U/mL) enhance platelet activation by forming optimal-sized immune complexes, whereas high concentrations (≥100 U/mL) disrupt complex formation and inhibit activation (1, 4). This biphasic heparin-response pattern is diagnostically characteristic.

In contrast, heparin-independent disorders (spontaneous HIT, VITT) are driven by antibodies that recognize PF4 complexed with endogenous polyanions other than heparin, such as glycosaminoglycans on endothelial surfaces, bacterial lipopolysaccharides, or viral components (2, 6, 10). Consequently, these antibodies activate platelets in the absence of exogenous heparin, and high-dose heparin often fails to inhibit-–or may paradoxically enhance-–activation in some cases (notably VITT). For aHIT specifically, the initial immunization relies on PF4-heparin immune complexes induced by exogenous heparin, whereas long-term platelet hyperactivation is maintained by monoclonal anti-PF4 clones that bind endogenous polyanion-PF4 complexes independent of circulating heparin. This two-stage pathogenic process distinguishes aHIT from spontaneous HIT-like syndrome and VITT, which never rely on heparin as an initial immunogen. Furthermore, the stoichiometry of PF4-polyanion complex formation may differ: in classic HIT, PF4 tetramers bind heparin in a highly ordered manner, whereas in heparin-independent forms, PF4 may interact with structurally heterogeneous polyanions (e.g., VWF, DNA, bacterial polysaccharides), leading to distinct antibody binding characteristics and pathogenic profiles (4, 11, 13). In addition to the polyclonal responses seen in classic HIT, aHIT is frequently driven by oligoclonal or monoclonal antibody populations. These antibodies arise from the expansion of specific B-cell clones, and somatic hypermutation within the variable regions of the immunoglobulin genes can confer enhanced binding affinity to PF4-polyanion complexes while simultaneously reducing dependency on heparin as a structural cofactor (22–24). This clonal nature explains the extreme refractoriness of aHIT to standard anticoagulation and the frequent requirement for immunomodulatory therapies such as IVIG or plasma exchange. These differences have direct therapeutic implications, explaining why high-dose IVIG (which blocks FcγRIIA) is more consistently effective in heparin-independent disorders, while anticoagulation alone often suffices in classic HIT.

#### 2.2.4 The role of PF4 tetramerization and stoichiometry in disease pathogenesis

PF4 is stored in platelet α-granules as a homotetramer (four 70-amino acid monomers) under physiologic conditions. This tetrameric structure is essential for its chemokine function and for its ability to bind polyanions. Each PF4 tetramer carries a highly positively charged surface (due to clustered lysine and arginine residues) that mediates electrostatic interactions with negatively charged polyanions such as heparin, chondroitin sulfate, bacterial lipopolysaccharide, and VWF (21).

In classic HIT, the binding of one heparin molecule to multiple PF4 tetramers creates an ultralarge immune complex that exposes neoepitopes recognized by pathogenic antibodies. The stoichiometry of PF4:heparin is critical: an optimal ratio (approximately 2:1 PF4 tetramers per heparin chain) yields maximal platelet activation (22). In heparin-independent disorders, the polyanion partners are structurally heterogeneous. For instance, PF4 binding to VWF may involve distinct binding interfaces and stoichiometry compared to PF4-heparin, potentially explaining why some anti-PF4 antibodies recognize PF4-VWF complexes but not PF4-heparin (21). Similarly, the binding of PF4 to bacterial lipopolysaccharide or viral RNA likely produces complexes with variable size and antigenicity (23). Evidence from our group and others suggests that these structural differences may influence antibody specificity, affinity, and pathogenic potential (21–24). Further studies are needed to define the precise stoichiometry and crystal structures of PF4 complexes with non-heparin polyanions.

### 2.3 Clinical features and diagnosis

A stepwise diagnostic workflow is recommended for suspected anti-PF4 disorders without proximate heparin exposure (1–15): 1) Clinical pre-test risk stratification via 4Ts score tailored for post-surgery, post-vaccination, infection and neonatal scenarios; 2) Initial serological screening using PF4-dependent ELISA; 3) Confirmatory functional platelet activation assays (SRA/PIPA) to distinguish pathogenic platelet-activating antibodies from non-pathogenic seropositivity; 4) Imaging verification of thrombosis; 5) Rigorous exclusion of mimicking syndromes including TTP, DIC, catastrophic APS and complement-mediated TMA (**Table 2** Laboratory and functional assay differences among anti-PF4 disorders).

Hematologic: Thrombocytopenia (The most common clinical manifestation, with platelet count usually below 150×10^9^/L, varying in severity. Some patients may experience severe thrombocytopenia (platelet count <20×10^9^/L)), elevated D-dimer (especially in VITT), variable fibrinogen.

Immunologic: PF4-dependent ELISA (preferred for VITT/spontaneous HIT). Rapid immunoassays may be falsely negative in heparin-independent forms.

Functional assays:

- Classic HIT: platelet activation enhanced by low heparin, inhibited by high heparin.
- VITT: activation inhibited by heparin; best detected by PF4-enhanced assays (PF4-SRA, PIPA).
- aHIT/spontaneous HIT:heparin-independent activation; refractory aHIT driven by monoclonal antibodies often exhibits sustained strong platelet activation regardless of heparin concentration in functional testing.

Imaging: To confirm thrombosis (CT, MRI, ultrasound).

Differential diagnosis (focused on thrombosis + thrombocytopenia):

- Thrombotic thrombocytopenic purpura (TTP): microangiopathic hemolytic anemia, not required pentad.
- Disseminated intravascular coagulation (DIC): underlying sepsis/trauma, global coagulation abnormalities.
- Antiphospholipid syndrome (APS)/catastrophic APS.
- Paroxysmal nocturnal hemoglobinuria (PNH).
- Complement-mediated TMA.

Isolated thrombocytopenia conditions (ITP, hypersplenism, myelosuppression) are usually not associated with acute thrombosis and should be excluded.

## 3 Specific subtypes of anti-PF4 disorders

### 3.1 Neonatal anti-PF4 disorders

True neonatal HIT-like syndrome is exceptionally rare. Most reported cases are due to transplacental transfer of maternal anti-PF4 antibodies, presenting within 24–48 hours after birth, sometimes with cerebral venous sinus thrombosis. *De novo* neonatal disease triggered by infection is possible but poorly documented. Treatment: IVIG, non-heparin anticoagulation if thrombosis present (25–27).

Diagnostic confirmation: Maternal serum should be tested alongside neonatal samples; persistent neonatal thrombocytopenia beyond 48–72 hours suggests *de novo* production rather than passive antibody transfer and warrants evaluation for infection or other etiologies.

For neonates with confirmed thrombosis, start non-heparin anticoagulation (argatroban, dosing adjusted for age and hepatic function) and consider high-dose IVIG (1 g/kg/day × 2 days) if severe thrombocytopenia (<50×10^9^/L) or progressive thrombosis. IVIG dosing in neonates should be based on ideal body weight, and fluid overload should be monitored closely.

### 3.2 Infection-associated anti-PF4 antibodies

Bacterial infections (e.g., Staphylococcus aureus, Streptococcus pneumoniae, Escherichia coli, Neisseria meningitidis) and viral infections (e.g., Epstein-Barr virus, human herpesvirus type 8, SARS-CoV-2, hantavirus) can induce the production of anti-PF4 antibodies (19, 28, 29). Mechanistically, PF4—a cationic protein—binds to negatively charged polyanions on bacterial cell walls (such as lipopolysaccharide and teichoic acid) or to viral components, forming immunogenic complexes that trigger an antibody response. This process is thought to represent an evolutionarily conserved component of the innate immune response against pathogens, facilitating opsonization and phagocytosis (23, 30). However, crucially, the vast majority of infection-induced anti-PF4 antibodies are non-platelet-activating and non-pathogenic. For example, in COVID-19 patients without heparin exposure, high-titer anti-PF4/heparin antibodies can be detected, yet most do not induce platelet activation (19). Similarly, in hantavirus-infected patients with hemorrhagic fever with renal syndrome, anti-PF4/heparin antibodies are common but correlate neither with disease severity nor with thrombocytopenia, indicating they are non-pathogenic bystanders (20). Rare, exceptional cases have been reported where infection is associated with true heparin-independent, platelet-activating anti-PF4 antibodies leading to thrombosis and thrombocytopenia, resembling VITT (so-called “VITT-like” disorders) (5). Nevertheless, such cases are not the norm. Therefore, clinicians should not assume pathogenicity based solely on anti-PF4 antibody positivity in the setting of infection. Functional testing (e.g., PF4-induced platelet activation assay or serotonin release assay) is required to determine whether antibodies have platelet-activating capacity, and alternative causes of thrombosis with thrombocytopenia (e.g., DIC, TTP, sepsis-associated coagulopathy) must be carefully excluded (19, 20, 23, 30).

In SLE or APS patients presenting with thrombocytopenia and thrombosis, anti-PF4 seropositivity should not be assumed pathogenic. Functional testing (SRA/PIPA) is mandatory before attributing the clinical picture to an anti-PF4 disorder. Alternative diagnoses—catastrophic APS, thrombotic microangiopathy, DIC—must be rigorously excluded. If functional assays confirm platelet-activating anti-PF4 antibodies, treatment follows the same principles as other heparin-independent disorders (non-heparin anticoagulation + IVIG), but concurrent immunosuppressive therapy for the underlying autoimmune disease should be maintained or adjusted in consultation with rheumatology.

### 3.3 Spontaneous HIT-like syndrome

Spontaneous HIT-like syndrome must not be conflated with autoimmune HIT (aHIT): unlike aHIT which requires prior heparin priming, this subtype develops thrombocytopenia and thrombosis with no history of heparin exposure at any time point, triggered solely by surgery, trauma or infection.

Spontaneous HIT-like syndrome is a distinct subtype of anti-PF4 antibody-mediated disease characterized by thrombocytopenia and thrombosis without recent heparin exposure. According to updated classification systems, it falls under the broader category of spontaneous HIT, which comprises two main subtypes (24–26): (a) post-surgical type (most commonly following total knee arthroplasty, cardiac surgery with cardiopulmonary bypass, or extracorporeal membrane oxygenation), and (b) post-infectious type. Compared with classic HIT, spontaneous HIT and other atypical anti-PF4 disorders (such as VITT) often present with more severe clinical courses, greater degrees of thrombocytopenia, higher frequencies of bleeding and thrombosis, and a predilection for unusual sites such as the cerebral venous sinuses and multiple vascular beds. Cases of spontaneous HIT following total knee arthroplasty have been reported in the literature, manifesting with clinical features highly similar to classic HIT despite no prior heparin exposure (31–33). Crucially, while spontaneous HIT arises without identifiable heparin exposure, aHIT is typically a sequelae of an initial heparin-triggered immune response that has evolved into a heparin-independent, often monoclonal, pathogenic state.

Diagnosis of spontaneous HIT-like syndrome requires integration of clinical assessment and laboratory testing. When clinical suspicion is high, the 4Ts score can be used for pre-test probability assessment. For immunological testing, PF4-dependent enzyme immunoassays (e.g., ELISA) are the most common screening tools, while functional platelet activation assays (e.g., PF4-SRA or PIPA) remain the gold standard for confirmation.

Regarding treatment, because spontaneous HIT-like syndrome is driven by persistent heparin-independent platelet-activating antibodies, immediate discontinuation of all heparin products and initiation of non-heparin anticoagulation (argatroban, bivalirudin, fondaparinux) remain fundamental measures (34, 35). However, unlike classic HIT, this syndrome frequently requires immunomodulation in addition to anticoagulation. High-dose intravenous immunoglobulin (IVIG, 1 g/kg/day for 2 days) is the preferred mechanism-based therapy, directly blocking FcγRIIA-mediated platelet activation, thereby rapidly improving platelet counts and reducing thrombotic risk (36, 37). Serial monitoring of platelet activation by functional serotonin release assays before and after IVIG can help assess treatment response and guide further management. For severe or refractory cases, therapeutic plasma exchange may be considered. The dual therapeutic strategy of “non-heparin anticoagulation plus high-dose IVIG” is dictated by the core pathophysiological mechanism of heparin-independent platelet-activating antibodies in spontaneous HIT-like syndrome.

### 3.4 VITT and VITT-like disorders

VITT is a well-defined entity following adenoviral vector COVID-19 vaccines (ChAdOx1, Ad26.COV2.S), incidence ~1/25, 000–1/250, 000, highest in young women (16). Onset 5–30 days post-vaccination. Key features: thrombocytopenia, thrombosis (often cerebral or splanchnic), markedly elevated D-dimer, low/normal fibrinogen, positive anti-PF4 ELISA, and platelet activation inhibited by heparin (in contrast to classic HIT) (17, 18). Early series reported mortality 20–50%, but more recent studies with prompt recognition and treatment show substantially improved outcomes. The proposed mechanism involves vaccine components (adenovirus) triggering PF4-dependent antibodies; molecular mimicry with spike protein is not strongly supported (38). Rare reports after other vaccines (influenza, HPV) exist but remain unproven and should not be generalized.

The NEJM review highlighted that VITT antibodies are associated with the IGLV3-21*02/*03 haplotype and a specific somatic hypermutation (K31E) that generates a negatively charged paratope enabling PF4 cross-reactivity (7). This genetic insight has direct clinical implications: (1) it explains why VITT is ultrarare despite widespread adenoviral vector vaccination, (2) it suggests that genetic testing may eventually inform risk stratification, though this remains investigational, and (3) it raises the possibility that similar stereotypic antibody responses may underlie other infection-triggered anti-PF4 disorders. Clinicians should be aware that VITT-like MGTS can occur without any vaccine or infection trigger, driven by a plasma cell clone producing monoclonal anti-PF4 antibodies; this entity should be suspected in patients with chronic or recurrent thrombocytopenia and thrombosis, particularly young patients, and serum protein electrophoresis/immunoelectrophoresis should be performed even when paraprotein levels are low.

### 3.5 Anti-PF4 antibodies in autoimmune diseases

Anti-PF4 antibodies can be detected in a minority of patients with systemic lupus erythematosus (SLE) and antiphospholipid syndrome (APS), but the overwhelming majority are non-platelet-activating and of uncertain pathogenic significance (2, 39). In SLE, heparin-dependent and heparin-independent anti-PF4 antibodies have been reported in 9% and 14% of patients, respectively, with heparin-independent antibodies correlating with disease activity, yet neither type induced platelet activation in functional assays (40). Similarly, false-positive HIT antigen tests are highly prevalent in APS/SLE (up to 76% in one series), but these antibodies universally fail to activate platelets by HIPA or aggregation tests, and they bind PF4 even in heparin excess, distinguishing them from true pathogenic HIT antibodies (41). Some studies have reported associations between anti-PF4 positivity and APS manifestations (e.g., arterial thrombosis), with partial cross-reactivity between anti-PF4 and IgM antiphospholipid antibodies (42), as well as the presence of anti-β_2_-GPI/PF4 complex antibodies in thrombotic APS (43). Nonetheless, consistent association with thrombosis has not been established. Thus, in autoimmune disease patients presenting with thrombocytopenia and thrombosis, anti-PF4 seropositivity alone does not prove pathogenicity; functional testing (e.g., PIPA or SRA) is required, and alternative diagnoses such as catastrophic APS, thrombotic microangiopathy, or DIC must be excluded. Rarely, truly pathogenic, heparin-independent anti-PF4 antibodies with platelet-activating capacity can occur in autoimmune contexts, as demonstrated in recent case reports (44, 45), but these are exceptional and should not be assumed without functional confirmation.

### 3.6 Drug-associated anti-PF4 immunothrombosis (rare) vs drug-induced thrombocytopenia (distinct)

True anti-PF4-mediated thrombosis with thrombocytopenia has been reported with vancomycin (vancomycin-induced thrombocytopenia, VIT) and a few other drugs, but this is rare (46, 47). Most drug-induced thrombocytopenias are due to hapten-dependent antibodies or bone marrow suppression and are not anti-PF4-mediated. These entities should not be conflated (47).

It is critically important to distinguish these genuine anti-PF4-mediated immunothrombotic events from the much more common forms of drug-induced thrombocytopenia (DIT) that are not mediated by anti-PF4 antibodies (46). Most DIT cases arise through hapten-dependent immune mechanisms, in which the drug or its metabolite binds covalently to platelet surface glycoproteins (e.g., GPIIb/IIIa), generating neoantigens that stimulate drug-dependent antiplatelet antibodies, leading primarily to isolated thrombocytopenia with bleeding risk rather than thrombosis (48, 49). Other non-immune mechanisms include direct myelosuppression (e.g., cytotoxic chemotherapies), drug-induced bone marrow suppression, or increased platelet destruction via non-immune pathways. Importantly, these entities—unlike anti-PF4-mediated disorders—do not typically present with the combination of thrombocytopenia and thrombosis. Therefore, clinicians should avoid conflating all drug-associated thrombocytopenias with anti-PF4 immunothrombosis; the presence or absence of concurrent thrombosis, along with appropriate laboratory testing for anti-PF4 antibodies and functional platelet assays, is essential for accurate diagnosis and appropriate management.

## 4 Treatment

The management of anti-PF4 disorders without proximate heparin exposure differs fundamentally from that of classic HIT, owing to the persistent heparin-independent platelet-activating properties of the pathogenic antibodies (9). In these entities, heparin cessation alone is insufficient, and immunomodulation is frequently required alongside anticoagulation. We propose a three-layer, risk-stratified therapeutic algorithm based on disease severity, subtype, and treatment response.

### 4.1 Acute management: a three-layer approach

#### 4.1.1 Layer 1: foundation therapy — immediate heparin cessation and non-heparin anticoagulation 

Rationale: Upon clinical suspicion of any anti-PF4 disorder without proximate heparin exposure, all sources of heparin (including intravenous flushes, heparin-coated catheters, and low-molecular-weight heparin) must be discontinued immediately. This recommendation applies regardless of the suspected subtype, for three reasons (2, 4–8, 11, 14, 15): (1) a mixture of antibody populations may coexist, and even low-dose heparin can exacerbate platelet activation via remaining heparin-dependent clones; (2) in VITT, the effect of heparin is variable rather than uniformly inhibitory across all concentrations; and (3) the initial differential diagnosis almost always includes classic HIT, which absolutely requires heparin withdrawal pending definitive subtyping.

Initiation Criteria (Layer 1 applies to ALL patients): Any patient with suspected anti-PF4 disorder without proximate heparin exposure (4Ts score ≥4 or strong clinical suspicion), pending confirmatory testing. **Table 3** summarizes the agents and dosing for layer 1-foundation therapy.

**Table 3:** Agents and dosing for layer 1-foundation therapy.

Agent	Dose	Renal adjustment	Hepatic adjustment	Monitoring
Argatroban	2 μg/kg/min IV (initial), titrate to aPTT 1.5–3× baseline	None	Reduce to 0.5–1.2 μg/kg/min in moderate-severe hepatic impairment	aPTT every 2–4 hours
Bivalirudin	0.15 mg/kg/hr IV (with PCI: 0.75 mg/kg bolus + 1.75 mg/kg/hr)	Reduce in CrCl <30 mL/min	None	aPTT or ACT
Fondaparinux	2.5 mg (prophylaxis) or 5–10 mg (treatment) SC daily based on weight	Avoid if CrCl <30 mL/min	None	Anti-Xa (optional)
Direct oral anticoagulants (DOACs)	Rivaroxaban 15 mg BID or Apixaban 5 mg BID (after platelet recovery >50×10^9^/L and hemodynamic stability)	Avoid if CrCl <30 mL/min	Avoid in Child-Pugh B/C	Clinical monitoring

Transition to oral anticoagulation: Switch to DOACs or warfarin only after platelet count has recovered to ≥50×10^9^/L (ideally ≥100×10^9^/L) and remains stable for ≥48 hours, with no evidence of new or progressive thrombosis (50, 51).

#### 4.1.2 Layer 2: adjunctive immunomodulation — high-dose intravenous immunoglobulin

Rationale: Unlike classic HIT, which often responds to anticoagulation alone, heparin-independent anti-PF4 disorders are driven by antibodies that activate platelets via FcγRIIA even in the absence of heparin. High-dose IVIG (1 g/kg/day for 2 consecutive days) competitively blocks FcγRIIA receptors, thereby interrupting pathogenic antibody-mediated platelet activation (11). This is a mechanism-based therapy rather than merely supportive care.

**Table 4** summarizes the specific indications for Layer 2, Dosing and Administration (52–56):

**Table 4:** Specific Indications for Layer 2 (initiate IVIG when any of the following criteria are met).

Clinical scenario	Indication strength	Supporting evidence
Confirmed VITT	**Strong** (first-line adjunct)	IVIG recommended as standard adjunct alongside anticoagulation
Confirmed aHIT with: (a) severe thrombocytopenia (<50×10^9^/L), (b) recurrent/progressive thrombosis despite adequate anticoagulation, (c) platelet count continuing to fall after heparin cessation	**Strong**	Case series demonstrate rapid platelet recovery and thrombosis resolution
Spontaneous HIT-like syndrome with life-threatening thrombosis (e.g., cerebral venous sinus thrombosis, splanchnic vein thrombosis) or platelet count <30×10^9^/L	**Moderate**	Recommended for severe/refractory cases
VITT-like MGTS with acute thrombotic exacerbation and falling platelet count	**Moderate**	IVIG provides temporary FcγRIIA blockade while definitive clone-directed therapy is initiated
Neonatal anti-PF4 disorder with confirmed thrombosis and severe thrombocytopenia (<50×10^9^/L)	**Moderate** (off-label)	Case reports support use; adjust for fluid balance

Bold-formatted entries represent priority clinical recommendations supported by case-series evidence and mechanistic rationale; randomized controlled trial data are lacking. Strong: IVIG is recommended for almost all patients meeting criteria. Moderate: IVIG should be individualized after risk-benefit assessment with hematology consultation.

Regimen: IVIG 1 g/kg ideal body weight/day for 2 consecutive days (total 2 g/kg).

Timing: Initiate as early as possible—preferably within 24–48 hours of diagnosis—as delayed administration is associated with poorer outcomes.

Pre-medication: Consider acetaminophen and diphenhydramine for infusion-related reactions; ensure adequate hydration to minimize renal risk.

Contraindications: IgA deficiency with anti-IgA antibodies (use IgA-depleted IVIG products if available); history of anaphylaxis to IVIG; uncompensated heart failure (use slower infusion rate and monitor fluid status).

Monitoring: Platelet count should be checked daily; a response is typically seen within 24–72 hours (rise ≥30×10^9^/L or stabilization). Functional platelet activation assays (SRA/PIPA) performed before and 3–5 days after IVIG can document blockade of antibody-mediated activation and guide further dosing if needed.

Repeat Dosing: If platelet count fails to improve or worsens after the initial 2-day course, a second course (1 g/kg/day × 2 days) may be considered in consultation with hematology/immunology, particularly in aHIT with monoclonal antibody persistence.

#### 4.1.3 Layer 3: rescue therapy — therapeutic plasma exchange

Rationale: TPE physically removes pathogenic anti-PF4 IgG antibodies, pro-inflammatory cytokines, and platelet-derived procoagulant microparticles. Unlike IVIG, which blocks FcγRIIA but leaves antibodies in circulation, TPE directly reduces antibody load (57, 58). However, TPE is resource-intensive, requires central venous access, and carries procedural risks; it is reserved for refractory or life-threatening cases.

**Table 5** summarizes the indications for Layer 3. TPE Protocol: Volume: 1–1.5 plasma volumes exchanged per session.

**Table 5:** Indications for Layer 3 (initiate TPE when any of the following criteria are met, preferably in a center with apheresis expertise).

Indication	Priority	Notes
Refractory to Layer 1 + Layer 2: platelet count <30×10^9^/L or progressive thrombosis despite ≥5 days of full-dose anticoagulation and one full course of IVIG (2 g/kg total)	**Highest**	TPE ± repeat IVIG after each session
Life-threatening thrombosis with multiorgan dysfunction (e.g., massive pulmonary embolism, extensive cerebral venous sinus thrombosis with hemorrhage, bilateral adrenal hemorrhage)	**High**	TPE as adjunct to anticoagulation + IVIG
Pre-operative bridging in aHIT patients requiring urgent heparin re-exposure (e.g., cardiac surgery)	**Moderate**	TPE can reduce antibody titers preoperatively, allowing safe intraoperative heparin use
VITT with refractory thrombocytopenia and D-dimer >20× upper limit of normal despite maximal medical therapy	**Moderate**	Limited case series support benefit

Bold-formatted entries represent priority rescue-therapy recommendations without randomized controlled trial support. Priority tiers: Highest, life‑threatening disease where delay risks major morbidity/mortality; High, significant end‑organ risk; Moderate, conditional/bridging indication for selected patients only.

Frequency: Daily for 3–5 sessions, then tapered based on clinical response and antibody titers.

Replacement fluid: 5% albumin is preferred; fresh frozen plasma is reserved for patients with significant coagulopathy (INR >1.8, fibrinogen <100 mg/dL).

Adjunctive IVIG: Administer IVIG (1 g/kg) immediately after each TPE session to replenish FcγRIIA blockade, as TPE removes both pathogenic antibodies and endogenous IgG.

Response Assessment: Measure platelet count daily and functional antibody titers (SRA/PIPA) before and after the TPE series. A rise in platelet count ≥30×10^9^/L or ≥50% from nadir, with stabilization or resolution of thrombosis, defines a positive response.

### 4.2 Investigational and future therapies

Several targeted agents are under investigation for anti-PF4 disorders. These remain experimental and should not be used outside of clinical trials or compassionate-use protocols.

#### 4.2.1 12-Lipoxygenase inhibitors

VLX-1005 is a first-in-class small-molecule inhibitor of 12-lipoxygenase (12-LOX), an enzyme involved in platelet activation downstream of FcγRIIA signaling, and the European Medicines Agency (EMA) has granted Orphan Drug Designation to VLX-1005 for the treatment of platelet-activating anti-PF4 disorders (59). VLX-1005 has successfully completed Phase 1 clinical trials, demonstrating a favorable safety and tolerability profile, and is currently enrolling patients in a Phase 2 trial for suspected HIT (NCT05785819) (60).

#### 4.2.2 Direct FcγRIIa receptor blockade

Given that FcγRIIa is the key receptor mediating platelet activation by anti-PF4 antibodies, direct blockade of this receptor represents a rational therapeutic strategy (60), which provide compelling evidence for developing FcγRIIa receptor blockers to treat VITT and other FcγRIIa-related thrombotic inflammatory disorders.

#### 4.2.3 Bruton’s tyrosine kinase inhibitors

For chronic anti-PF4 disorders such as VITT-like MGTS, BTK inhibitors (e.g., ibrutinib, pirtobrutinib) target the B-cell clone producing the pathogenic monoclonal paraprotein. Case reports have shown efficacy in reducing paraprotein levels and preventing recurrent thrombosis (58, 59, 61). This approach is disease-modifying rather than merely symptom-controlling, and represents a promising direction for chronic management, though prospective trials are lacking.

## 5 Conclusion

This review systematically summarizes three core subtypes of anti-PF4 disorders without proximate heparin exposure: heparin-primed autoimmune HIT (aHIT), surgery/infection-triggered spontaneous HIT-like syndrome, and adenovirus vaccine-induced VITT/TTS. Beyond consensus pathogenic pathways shared with prior literature, this work highlights our team’s original PF4-VWF immunogenic complex hypothesis to explain non-heparin anti-PF4 immunothrombosis. Furthermore, we constructed scenario-specific stratified diagnostic workflows and tiered therapeutic strategies (anticoagulation + first-line IVIG + rescue plasma exchange) for neglected vulnerable subgroups including neonates, orthopedic postoperative patients, and individuals with underlying autoimmune disease. Future research priorities include standardized subtype-specific diagnostic criteria, PF4-VWF complex-based novel biomarkers, and clinical validation of FcγRIIA/12-LOX targeted therapies for heparin-independent anti-PF4 disorders.

From a clinical standpoint, the key takeaway is that anti-PF4 antibody positivity does not equal pathogenicity, particularly in infection or autoimmune settings, where non-activating antibodies are the rule rather than the exception. Functional testing remains the cornerstone for differentiating pathogenic from bystander antibodies. Furthermore, given the rarity of these heparin-independent disorders, multicenter registries and international collaborations are urgently needed to validate diagnostic criteria and generate high-level evidence for targeted therapies, particularly in Asian populations where vaccine platforms and infectious disease spectra differ substantially from Western cohorts.

Several important limitations merit acknowledgment. First, the proposed PF4-VWF pathogenic complex hypothesis remains speculative and has not been validated in animal models or human prospective cohorts; its role in clinical anti-PF4 disorders without heparin exposure requires confirmation through structural biology studies (e.g., cryo-EM of PF4-VWF complexes) and functional neutralization experiments. Second, the diagnostic and therapeutic algorithms presented are largely extrapolated from classic HIT literature and expert consensus rather than randomized controlled trial data, which are lacking for all heparin-independent anti-PF4 disorders. Third, while we provide stratified recommendations for special populations (neonates, autoimmune disease patients, post-surgical patients), the evidence base for each subgroup is limited to case reports and small case series, and our suggested algorithms should be validated in larger, preferably multicenter, cohorts before widespread adoption. Fourth, the relative rarity of these disorders (particularly VITT and spontaneous HIT-like syndrome) precludes high-level evidence for comparative effectiveness of different anticoagulants or the optimal timing and dosing of IVIG; our recommendations reflect current expert opinion and may evolve as more data emerge. Fifth, this review focuses predominantly on Western literature and may not fully capture the epidemiology, trigger profiles, or genetic predispositions (e.g., the IGLV3-21*02/*03 haplotype) relevant to Asian populations, where adenoviral vector vaccine usage, infectious disease spectra, and heparin prescribing patterns differ substantially. International collaborative registries are urgently needed to address these gaps.
